# Supplementary material for: Causal Relationship between Aging and Anorexia Nervosa: A White-Matter-Microstructure-Mediated Mendelian Randomization Analysis
Source: Biomedicines. 2024 Aug 16;12(8):1874. doi: 10.3390/biomedicines12081874 (PMC11351342; doi:10.3390/biomedicines12081874)
Supplement: Supplementary file 1 [file biomedicines-12-01874-s001.zip › Supplementary Figure/Figure S4/Singe SNP plot of MR analysis for TL to ubm-b-1920.pdf]

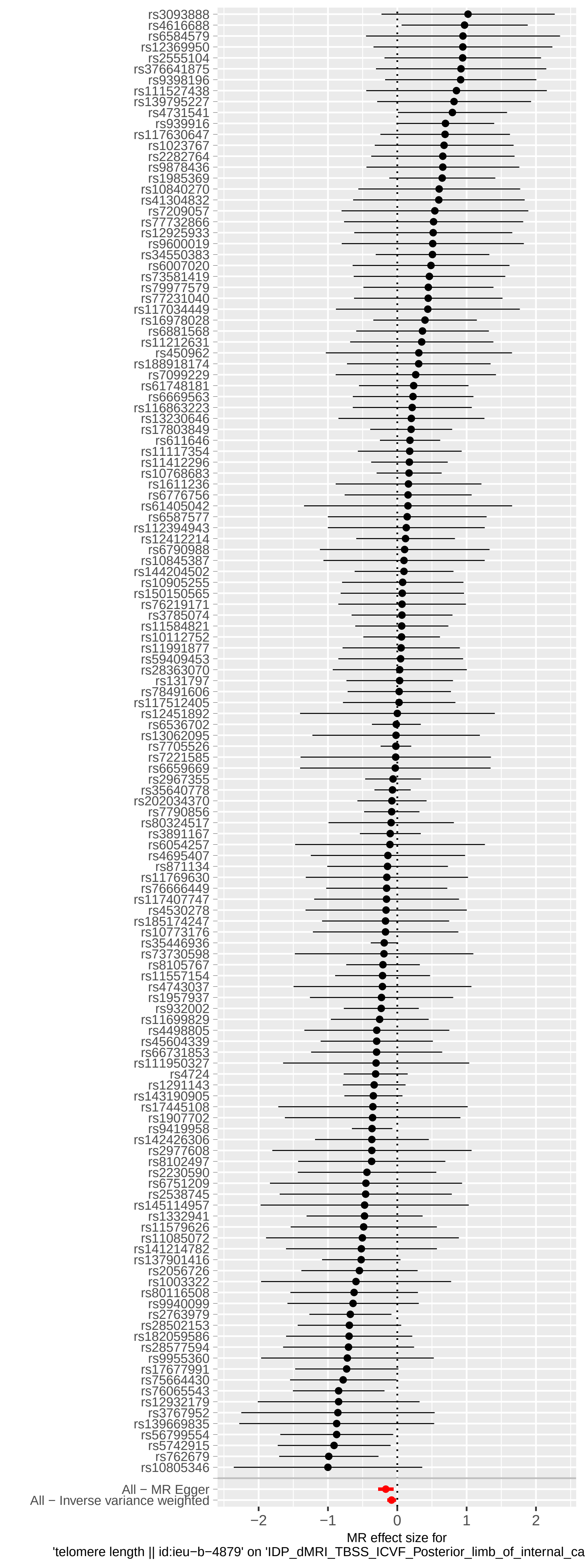
rs3093888
rs4616688
rs6584579
rs12369950
rs2555104
rs376641875
rs9398196
rs111527438
rs139795227
rs4731541
rs939916
rs117630647
rs1023767
rs2282764
rs9878436
rs1985369
rs10840270
rs41304832
rs7209057
rs77732866
rs12925933
rs9600019
rs34550383
rs6007020
rs73581419
rs79977579
rs77231040
rs117034449
rs16978028
rs6881568
rs11212631
rs450962
rs188918174
rs7099229
rs61748181
rs6669563
rs116863223
rs13230646
rs17803849
rs611646
rs11117354
rs11412296
rs10768683
rs1611236
rs6776756
rs61405042
rs6587577
rs112394943
rs12412214
rs6790988
rs10845387
rs144204502
rs10905255
rs150150565
rs76219171
rs3785074
rs11584821
rs10112752
rs11991877
rs59409453
rs28363070
rs131797
rs78491606
rs117512405
rs12451892
rs6536702
rs13062095
rs7705526
rs7221585
rs6659669
rs2967355
rs35640778
rs202034370
rs7790856
rs80324517
rs3891167
rs6054257
rs4695407
rs871134
rs11769630
rs76666449
rs117407747
rs4530278
rs185174247
rs10773176
rs35446936
rs73730598
rs8105767
rs11557154
rs4743037
rs1957937
rs932002
rs11699829
rs4498805
rs45604339
rs66731853
rs111950327
rs4724
rs1291143
rs143190905
rs17445108
rs1907702
rs9419958
rs142426306
rs2977608
rs8102497
rs2230590
rs6751209
rs2538745
rs145114957
rs1332941
rs11579626
rs11085072
rs141214782
rs137901416
rs2056726
rs1003322
rs80116508
rs9940099
rs2763979
rs28502153
rs182059586
rs28577594
rs9955360
rs17677991
rs75664430
rs76065543
rs12932179
rs3767952
rs139669835
rs56799554
rs5742915
rs762679
rs10805346
All − MR Egger
All − Inverse variance weighted
−2
−1
0
1
2
MR effect size for
'telomere length || id:ieu−b−4879' on 'IDP_dMRI_TBSS_ICVF_Posterior_limb_of_internal_ca
